# Auditory speed processing in sighted and blind individuals

**DOI:** 10.1371/journal.pone.0257676

**Published:** 2021-09-22

**Authors:** Giorgia Bertonati, Maria Bianca Amadeo, Claudio Campus, Monica Gori

**Affiliations:** 1 Unit for Visually Impaired People (U-VIP), Istituto Italiano di Tecnologia, Genova, Italy; 2 Department of Informatics, Bioengineering, Robotics and Systems Engineering (DIBRIS), Università degli Studi di Genova, Genova, Italy; University of Montreal, CANADA

## Abstract

Multisensory experience is crucial for developing a coherent perception of the world. In this context, vision and audition are essential tools to scaffold spatial and temporal representations, respectively. Since speed encompasses both space and time, investigating this dimension in blindness allows deepening the relationship between sensory modalities and the two representation domains. In the present study, we hypothesized that visual deprivation influences the use of spatial and temporal cues underlying acoustic speed perception. To this end, ten early blind and ten blindfolded sighted participants performed a speed discrimination task in which spatial, temporal, or both cues were available to infer moving sounds’ velocity. The results indicated that both sighted and early blind participants preferentially relied on temporal cues to determine stimuli speed, by following an assumption that identified as faster those sounds with a shorter duration. However, in some cases, this temporal assumption produces a misperception of the stimulus speed that negatively affected participants’ performance. Interestingly, early blind participants were more influenced by this misleading temporal assumption than sighted controls, resulting in a stronger impairment in the speed discrimination performance. These findings demonstrate that the absence of visual experience in early life increases the auditory system’s preference for the time domain and, consequentially, affects the perception of speed through audition.

## Introduction

Humans interact with moving sounds in their surrounding environment. The ability to track acoustic sources’ motion allows individuals to predict movement outcomes and guide their actions accordingly. For example, humans can avoid an approaching car in the street by processing the motion of an engine roar. This auditory ability becomes even more crucial when the visual stimulus cues are unavailable, such as if the car is approaching from behind a corner and one can only hear the sound it is producing. The acoustic motion may be described as a change of sound location over time. When interacting with a moving stimulus, the brain extracts both spatial and temporal information, computing the speed at which the sound is moving. Some scientific studies on auditory motion focus precisely on acoustic speed perception [1–4]. Freeman and colleagues [4] revealed that temporal cues dominate the auditory motion perception when interacting with moving sounds. Listeners are more sensitive to changes in the duration of the sound than to changes in its distance or actual speed (i.e., the ratio between space and time). Nevertheless, when adding noise to make the motion temporal components irrelevant (for details on the noise masking technique, see [5, 6]), listeners begin to rely also on speed cues, suggesting that, when forced, the auditory system can still encode velocity and process the interaction between spatial and temporal aspects [4]. Overall, these results outline that speed is not a primary component in the auditory system and challenge the existence of acoustic motion-sensitive detectors similar to those found in vision. In this regard, past studies have identified direction and velocity detectors in the visual system, with observers processing moving stimuli based on the ratio between space and time, i.e. the speed of the stimulus [5–7]. On the contrary, other studies have suggested that audition lacks low-level motion detectors that respond selectively to velocity and direction information. Indeed, the auditory system reconstructs acoustic motion by using binaural (interaural-time differences, ITDs, and interaural-level differences, ITLs) and monoaural (filtering properties of the pinna) cues that it detects over time [8, 9].

When speaking about motion processing, we must consider that different sensory modalities have a specific predisposition for representing the spatial or temporal domain–aspects intrinsically involved in motion processing. For example, previous studies outlined that audition has a superior ability for treating temporal information [10, 11] and vision when encoding spatial information [12]. Sensory impairment demonstrates the essential role of these sensory signals in creating space and time representations. For what concerns the lack of audition, past studies revealed impaired performance in constructing complex temporal metrics in deaf adults [13] and deaf children with restored hearing [14], suggesting that auditory experience crucially influences temporal representation development. In other studies, deaf individuals reported similar temporal perceptual thresholds to controls ([15, 16]; for a review see [17]). Regarding lack of vision and how temporal and spatial abilities develop in blindness, while good performance is maintained in the temporal domain [18–22], conflicting results have emerged about spatial skills. On the one side, blindness has been associated with enhanced auditory spatial abilities [23–25], possibly related to cortical plasticity mechanisms [26, 27]. On the other side, blind individuals reported impaired spatial skills [21, 22, 28, 29] that have been explained by a fundamental role of vision in the calibration of the different sensory modalities for spatial representations. Moreover, previous studies demonstrated that if vision is unavailable, a person uses the temporal properties of an acoustic event to infer its spatial information, suggesting that blind individuals preferentially rely on temporal cues when processing spatial representations they are not able to solve [30, 31].

By focusing on auditory motion perception, some past studies have shown that individuals with visual disabilities can detect the motion of a sound source [32–38] and present with an enhanced ability in judging the direction of moving sounds [39]. However, another study [40] investigating a more complex motion structure (a sound moving in different trajectories within a circle) showed that blind people are impaired when tracking sound movement. Therefore it is still unclear if the absence of vision promotes an enhanced motion perception or if the lack of a visual calibration determines an impaired auditory motion perception. In this debate, one specifically unclear aspect is how blindness influences the use of motion cues by the auditory system. Two possible mechanisms might act in the absence of vision. On the one side, the visual speed detectors revealed in the sighted individuals might be recruited by audition in the blind population, leading to a better acoustic speed-tuned performance compared to sighted people. On the other side, this cortical reorganization of motion detectors might not occur and the performance of blind individuals may be characterized by a peculiar use of temporal information during motion perception. In the present study, we leant towards the second point of view. Indeed, the difficulties of people with visual disabilities in dealing with spatial information, together with the specialized support they receive from the temporal domain [30, 31], suggested that the blind individuals’ ability of tracking sound movements may be influenced by a peculiar processing of spatio-temporal information for auditory motion perception.

In light of these considerations, we investigated how spatial and temporal information is used for sounds’ speed discrimination in the lack of vision, to test this hypothesis. Ten early blind and ten sighted individuals performed a speed discrimination task in which spatial, temporal, or both cues were available to infer the velocity of moving sounds. We expected a temporal dominance during speed processing in both sighted and blind individuals, and we hypothesized that this temporal preference influenced, even more, the speed discrimination performance of people with visual impairment.

## Material and methods

### Participants

A group of 10 early blind participants (EB, 6 females, mean age ± SD: 38.22 ± 11.39 y.o., range 22–55 y.o.) and 10 age-matched sighted controls (SC, 5 females, 38.99 ± 10.83 y.o., range 25–52 y.o.) took part in the experiment. Regarding EB individuals, visual impairment was attributed to peripheral deficits (i.e., deficits of the retina or optic tract), and blindness was total except for light perception in three participants and light and shape perception in two (for clinical details, see Table 1). None of the participants reported additional sensory disabilities or neurological problems. The study was approved by the ethics committee of the local health service (Comitato Etico, ASL3 Genovese) and conducted following the Declaration of Helsinki. All individuals gave written informed consent before starting the test.

**Table 1:** Clinical details of early blind participants.

Participant	Age	Gender	Pathology	Blindness onset	Residual vision
EB1	48	M	Atrophy optic nerve	Birth	No vision
EB2	22	F	Retinopathy of prematurity	Birth	Light and shadows
EB3	32	F	Retinitis pigmentosa	Birth	1/20 visual acuity.
					<1% visual field.
					Light and shapes
EB4	31	F	Retinopathy of prematurity	Birth	No vision
EB5	44	F	Retinitis pigmentosa	Birth	1/50 visual acuity.
					Light and shapes
EB6	33	F	Retinopathy of prematurity	Birth	No vision
EB7	28	M	Leber amaurosis	Birth	No vision
EB8	55	M	Retinopathy of prematurity	Birth	Light and shadows
EB9	54	M	Unknown	Birth	No vision
EB10	32	F	Retinitis pigmentosa	Birth	Light and shadows

EB: early blind, M: male, F: female.

### Setup, stimuli and procedure

Participants were sitting 200 cm away from the center of an array of 24 speakers spanning 50° of visual angle. Auditory stimuli consisted of moving white noises (10000 Hz, 80 dB at ears’ level). The acoustic motion was simulated by reproducing sequential white noises along with the array of speakers and modulating the volume of the sounds shaping a Gaussian distribution. Acoustic stimuli moved either rightward or leftward in a randomized order to prevent participants’ expectations. For each motion direction, they could randomly begin from the most extreme speaker of the array or from the two speakers immediately next to it.

**Fig 1:**
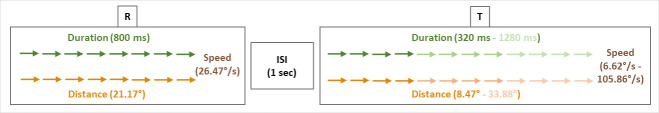
Illustration of a trial: The reference sound (R) moved at the fixed speed of 26.47°/s, the target sound (T) at faster or slower speeds according to the manipulation of the reference distance and/or duration. R and T were always separated by 1 second interstimulus interval (ISI).

**Fig 2:**
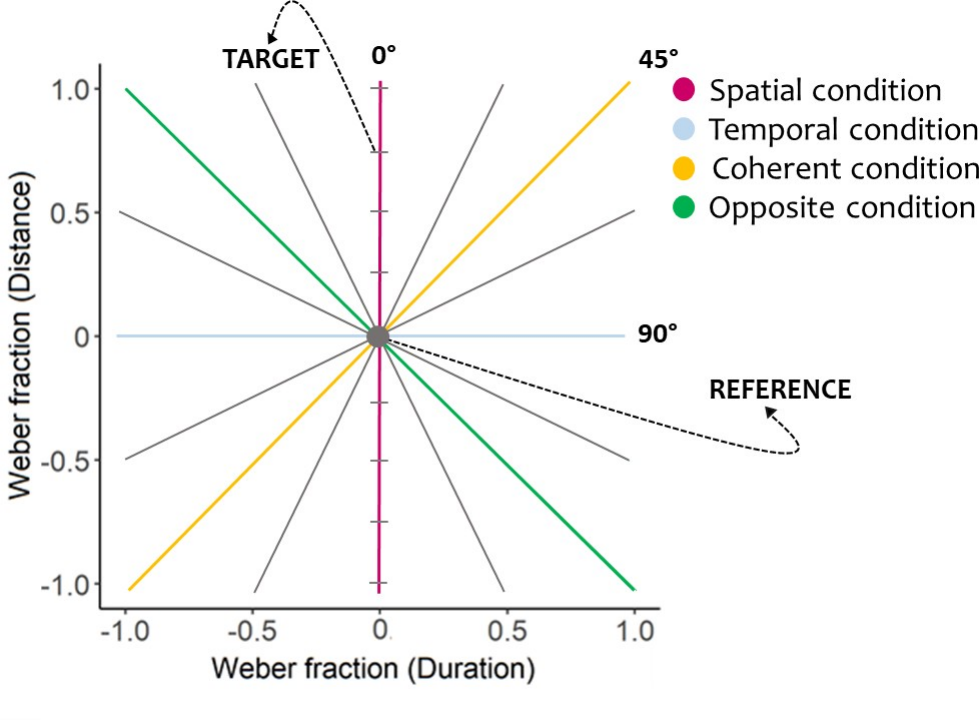
Distance-duration plane: The eight lines composing the plane correspond to eight orientations. Along with each orientation, the target sound (here selected as an example) differed from the reference sound (the grey dot) by a given proportion of spatial (Distance) and temporal (Duration) cues. Among the eight orientations, the colored lines correspond to the four experimental conditions.

Each trial consisted of a reference sound (R) moving at a fixed speed of 26.47°/s (traveled distance: 21.17°; duration: 800 ms), and a target sound (T) moving at different speeds ranging from 6.62°/s to 105.86°/s (Fig 1). More specifically, we computed the target sound’s speeds by manipulating the stimulus’ duration and travelled distance so that the target differed from the reference by given proportions (Weber fraction, W) of the reference duration and distance. In particular, $W_{distance}=\frac{\left(T_{distance}-R_{distance}\right)}{R_{distance}}$ and $W_{duration}=\frac{\left(T_{duration}-R_{duration}\right)}{R_{duration}}$, where *distance* refers to the spatial displacement of the stimulus along with the array (in degrees) and *duration* to the stimulus’s temporal length (in ms). Fig 2 presents the resulting set of target speeds in the distance-duration plane. From each orientation of the plane (see the eight lines reported in Fig 2), we selected nine target speeds via a constant stimuli method and repeated them six times each in a randomized order for a total of 432 trials.

To evaluate the role of spatial and temporal cues in speed discrimination performance, we selected four experimental conditions among the orientations of the distance-duration plane (see the four colored lines in Fig 2): i) a spatial condition where only distance cues were manipulated; ii) a temporal condition where only duration cues were manipulated; iii) a coherent condition where distance and duration cues were manipulated with a directly proportional relationship, and iv) an opposite condition where distance and duration cues were manipulated with an inversely proportional relationship. In the four experimental conditions, speed changed according to manipulation of duration and/or distance: i) in the spatial condition, when distance increased, speed increased; ii) in the temporal condition, when duration increased, speed decreased; iii) in the coherent condition, when distance increased, duration increased proportionally, thus speed was constant (i.e., the target sound speed was equal to the reference sound speed at any plane orientation point); iv) in the opposite condition, when distance increased, duration decreased, thus speed increased (i.e., speed was fast for short durations/long distances and slow for long durations/short distances).

All participants performed a two-alternative forced-choice (2AFC) task in which, for each trial, they had to determine the speed of the target sound by saying whether it was moving faster or slower than the reference sound. According to the experimental condition, participants could rely on spatial, temporal, or both cues to identify the target sound’s speed (see Fig 2). Participants supplied their answers by pressing one of two keys corresponding to "faster" or "slower" perception. No feedbacks were given to participants during the task. The experiment was divided into 6 blocks with randomly selected target speeds.

Participants were asked to keep their heads steady while performing the task, with the experimenter constantly monitoring the subjects’ head position. Sighted individuals and the two participants with residual light and shape perception (see Table 1 for details) were blindfolded before entering the room, which ensured that they could not see the setup throughout the experiment.

### Data analysis

**Fig 3:**
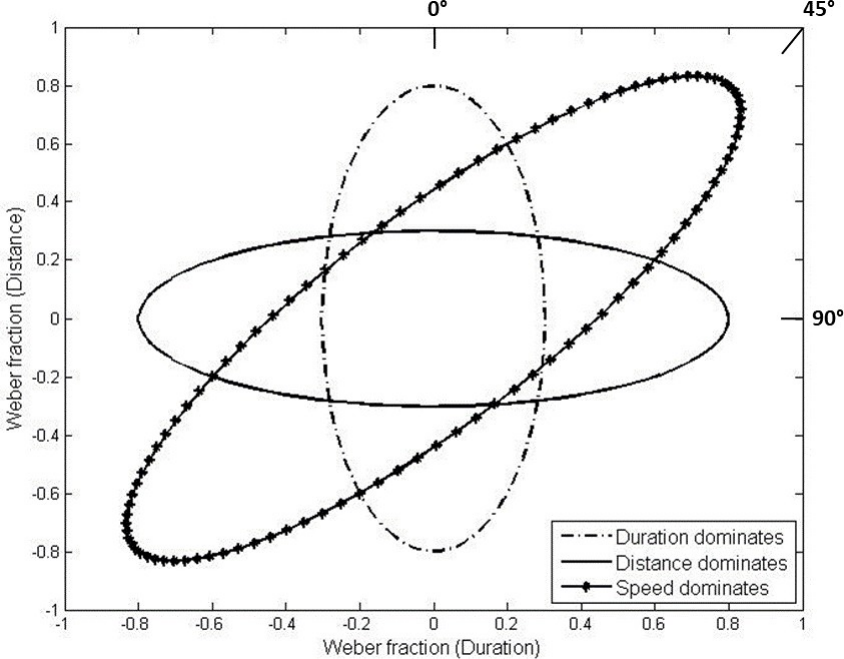
Exemplar ellipse’s orientations: Three exemplar ellipses have been applied to the distance-duration plane resembling the three dominant orientations: Duration-dominant orientation (0°), speed-dominant orientation (45°) and distance-dominant orientation (90°). These ellipses’ orientations would suggest if participants are more sensitive towards temporal, speed or spatial cues of auditory motion.

We analyzed data using a discrimination contours technique to disentangle the contribution of spatial and temporal information in the speed task. Other studies applied this method to differentiate the perceptual components underlying color vision and motion perception processes [4–7, 41]. More specifically, we calculated the proportion of "faster" respon432ses for each participant as a function of W_distance_ and W_duration_ in two separate analyses. For each orientation of the distance-duration plane, psychometric curves were fitted to these two proportions by cumulative Gaussian function. For each subject, a total of 16 just noticeable difference (JND) scores were extracted from the standard deviation of the best fitting function (8 from the W_distance_ fit and 8 from the W_duration_ fit). The set of 16 JNDs of each subject was then applied as a Cartesian coordinate system to the distance-duration plane, and ellipses were fitted to the set by the function *fit_ellipse* in Matlab [42]. The sets of JNDs of four participants (3 EB and 1 SC) were not suitable for ellipses’ fitting with this function. We, therefore, excluded these subjects from the ellipse orientation analysis only. Nevertheless, visual inspection of these sets of JNDs reveled elliptical-shaped plots similar to the performance profile of the others participants, supporting a consistency of the results also in the excluded subjects. We interpreted the ellipse orientations according to the discrimination contours technique, expecting ellipses oriented along with the oblique orientation (45°, *speed-dominant orientation*) when participants preferentially encoded speed cues (that include both spatial and temporal information), ellipses oriented parallel to the y-axis (0°, *duration-dominant orientation*) when they preferentially encoded temporal cues, and ellipses oriented parallel to the x-axis (90°, *distance-dominant orientation*) when they preferentially encoded spatial cues. Thus, for example, large JNDs when only spatial information was provided (in the spatial condition) and small JNDs when only temporal information was provided (in the temporal condition) resulted in a vertical ellipse oriented along with the duration-dominant orientation. It means that participants, in the speed discrimination, were more sensitive to the temporal cues rather than to the distance or the speed cues. For a graphical explanation of ellipses’ outcomes see also Fig 3. To statistically evaluate if SC and EB groups were more sensitive to spatial, temporal, or speed information, we compared the orientation of the subjects’ ellipses (in degrees) to the three dominant orientations (0°, 45°, 90°). Data were not normally distributed for one group, as verified with Shapiro–Wilk tests (EB: W = 0.86, p-value = 0.008; SC: W = 0.93, p-value = 0.495), so we ran three permutation paired t-tests per group through the *perm*.*t*.*test* function for R (*RVAideMemoire* package, https://cran.r-project.org/package=RVAideMemoire).

**Fig 4:**
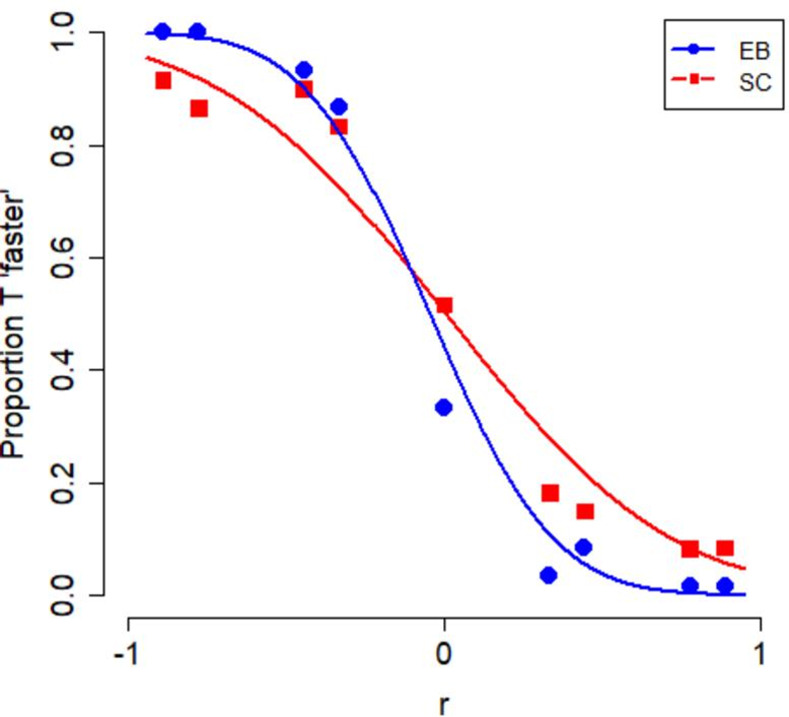
Psychometric curves of a sighted participant (red curve) and an early blind participant (blue curve): Exemplifications of inverted psychometric curves, fitted to the proportion of “faster” responses on the radial distance (r). Inverted psychometric curves denoted an impediment in performing the speed discrimination task. In this example, the reported psychometric curves refer to the coherent condition.

The proportion of "faster" responses was successively calculated as a function of the radial distance $r=\sqrt{\left(W_{{distance}^{2}}+W_{{duration}^{2}}\right)}$ [4] to evaluate the ability to discriminate speed through audition for each condition. This value is an index of the distance of the target sound from the reference sound and the distance of target spatial and temporal information composing the speed percept. When *r* = 0, target and reference speeds were equal. *r* values associated with points on the plane with y coordinates < 0, were turned to negative values (*r*_*turned*_ = -*r*) [4, 41]. Data were fitted by cumulative Gaussian functions and JND scores extracted to measure the subject’s precision. We verified each psychometric function’s goodness of fit by checking the significance of its R squared at alpha level = 0.05. When it was not significant, we assigned the worst JND of the sample to the function. Previous studies have already validated this technique [13, 43], driving us to interpolate four SC participants in the spatial condition. It is also worth noting that both groups produced inverted psychometric curves in the spatial and coherent conditions corresponding to negative JNDs (an example in Fig 4). This change was because participants did not base their answers on the stimulus’s speed features, but rather only on its spatial or temporal cues. This mechanism led them to fail to perform the speed discrimination task. To include these conditions together with the others, we decided to apply a conversion to the negative JND (JND_neg_), where JND_conv_ = JND_neg_—min (JND) + max (JND) for each group [13, 31]. After this conversion, low JND_conv_ meant good precision in speed discrimination, and high JND_conv_ meant poor precision in speed discrimination. At the same time, high JND_conv_ meant good precision in either the temporal or the spatial domain, depending on the condition. To compare the two experimental groups in their ability to discriminate the speed of moving sounds, we performed a permutation ANOVA on JND_conv_ values. The dependent variable was not normally distributed for one group (Shapiro-Wilk normality tests: EB: W = 0.91, p-value = 0.007; SC: W = 0.97, p-value = 0.386), we then ran a permutation test with Group (EB vs SC) as between-subjects factor, and Condition (spatial vs temporal vs coherent vs opposite) as a within-subjects factor. We used the *aovperm* function for R (*permuco* package, https://cran.r-project.org/package=permuco) to compute the analysis, running five thousand iterations.

Alpha level was set at 0.05 for all statistical analyses and Bonferroni correction used for multiple comparisons.

## Results

**Fig 5:**
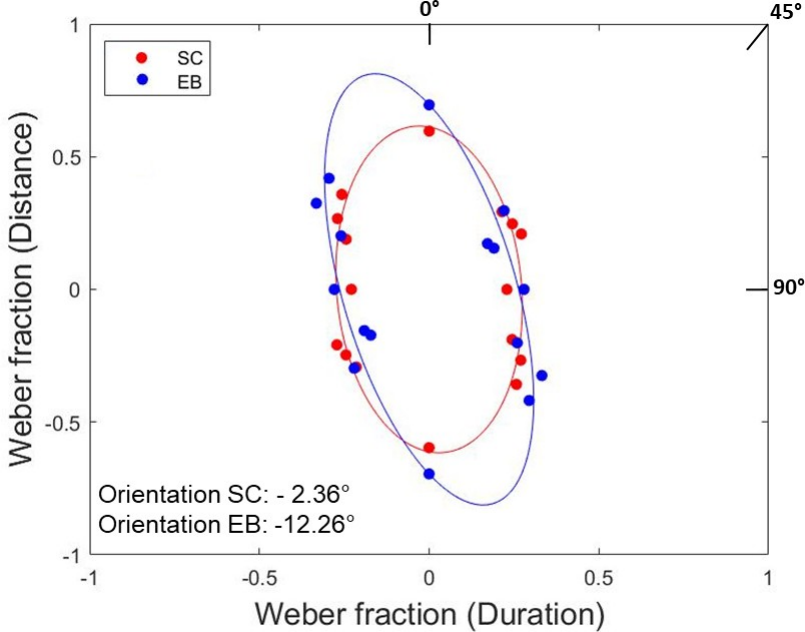
Ellipses of EB (in blue) and SC (in red) groups: For each ellipse, data points represent the JNDs of the psychometric functions plotted along each orientation. Ellipses oriented close to the 0° axis suggest temporal dominance.

The present study evaluates how early blind and sighted participants combined spatial, temporal and speed cues in perceiving auditory motion. Fig 5 shows the best-fitting ellipses of EB and SC participants. Ellipses of the two groups are oriented vertically, which shows that both EB and SC were more sensitive to changes in stimuli temporal duration than travelled distance or speed (for a graphical example of ellipse’s orientation see Fig 3). This specialized mechanism suggests that, in the task, spatial and temporal information were separable dimensions and that temporal processing cues dominated speed discrimination performance. Statistical analysis also confirmed temporal dominance. For both groups, permutation paired t-tests showed that the orientation of the ellipses was not significantly different from *duration-dominant orientation* (0°: SC: t = -1.28, p-value = 0.234, 95% CI [-1.05, 0.26], Hedges’s g = -0.39; EB: t = -2.55, p-value = 0.078, 95% CI [-1.74, -0.03], Hedges’s g = -0.84). On the contrary, ellipses were oriented away from the *distance-dominant orientation* (90°: SC: t = -27.57, p-value = 0.005, 95% CI [-12.89, -4.52], Hedges’s g = -8.30; EB: t = -20.21, p-value = 0.015, 95% CI [-11.18, -3.19], Hedges’s g = -6.64) and the *speed-dominant orientation* (45°: SC: t = -14.43, p-value = 0.002, 95% CI [-6.90, -2.31], Hedges’s g = -4.34; EB: t = -11.38, p-value = 0.015, 95% CI [-6.36, -1.17], Hedges’s g = -3.74). Finally, EB and SC participants did not differ in terms of ellipses orientation (t = 1.46, p-value = 0.178, 95% CI [-1.74, 0.35], Hedges’s g = -0.69). Overall, these results suggest that participants relied less on speed cues (i.e. ratio between spatial and temporal cues) and preferred to base their judgments on the temporal components of moving sounds (temporal dominance).

**Fig 6:**
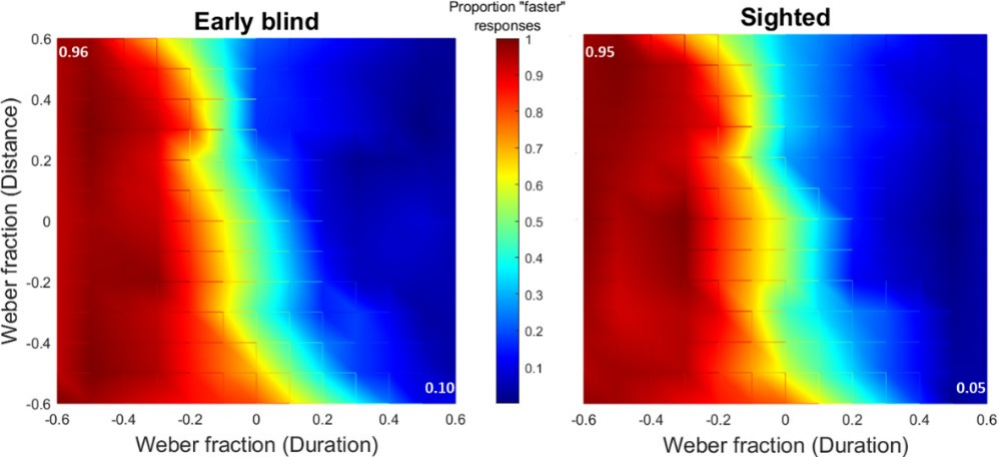
“Faster” proportion graph: On the distance-duration plane, the proportion of “faster” responses was plotted for each point of the plane, for the two groups separately. On the left-up and right-down corners of each graph, “faster” proportion values were reported.

Further qualitative analyses on participants’ temporal dominance revealed that sighted and blind individuals, in discriminating the auditory speed, also followed a temporal assumption that identified as faster those sounds with a shorter duration. The graphs in Fig 6 show subjects’ proportion of "faster" responses for each point of the distance-duration plane. High "faster" proportions localized on the left side of the graphs suggest that participants identified target sounds that lasted less than the reference sound as faster stimuli. Moreover, by comparing the proportion for the longer duration/shorter distance points (lower-right corner) with the shorter duration/longer distance points (upper-left corner), we revealed that this mechanism preferentially referred to the temporal encoding of the stimulus rather than to its spatial dimension. For both groups, “faster” proportion was significantly higher in the shorter duration/longer distance point than in the longer duration/shorter distance point (EB: χ^2^(1,10) = 87.09, p-value < 0.001, 95% CI [0.32, 0.53], Cramer’s V = 0.43; SC: χ^2^(1,10) = 93.63, p-value < 0.001, 95% CI [0.30, 0.52], Cramer’s V = 0.41). This result means that the speed was considered faster every time the target sound was shorter in time independently of space (temporal assumption), but not every time it was shorter in space. We soon realized that, in some circumstances, participants became unable to perform the speed discrimination task by following this temporal assumption. More specifically, in the coherent condition, the temporal assumption prevented participants from perceiving that the target speed was equal to the reference speed. In the spatial condition, the temporal assumption prevented participants from recognizing that shorter distances corresponded to slower speeds (for a detailed explanation of spatial, temporal and speed parameters in each condition, see the Method section).

**Fig 7:**
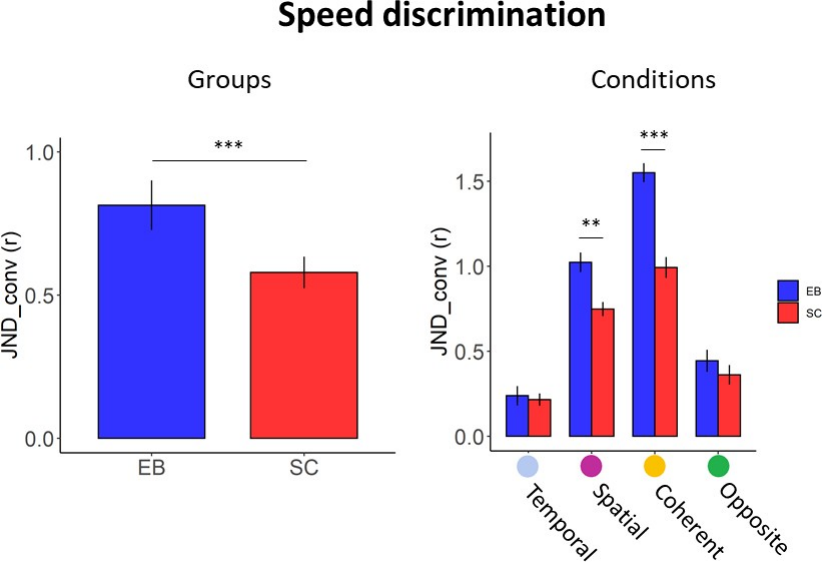
Group performance in the speed discrimination task: The left panel reports the average JND_conv_ of each group. The right panel reports the average JND_conv_ per group for each experimental condition. Error bars show standard errors. Asterisks indicate significance level: ** p < 0.01, *** p < 0.001.

We then compared the two groups in their speed discrimination precision (JND_conv_) based on the influence of visual experience on discriminating speed through audition. Permutation ANOVA on JND_conv_ revealed a significant main effect of Group (Iter: 5000, F = 97.69, p-value < 0.001, 95% CI [0.19, 0.15], partial *η*^2^ = 0.36), with EB participants showing a lower precision in determining the speed of the moving sounds compared to SC (Fig 7). A main effect of Condition (Iter: 5000, F = 182.34, p-value < 0.001, 95% CI [0.33, 0.62], partial *η*^2^ = 0.50) highlighted that, for both SC and EB, when only temporal cues were available to discriminate the speed of the moving sounds (temporal condition), the precision in the task was higher compared to all the other conditions (spatial: t = -11.48, p-value < 0.001, 95% CI [-3.11, -1.43], Hedges’s g = -2.22; coherent: t = -12.66, p-value < 0.001, 95% CI [-3.45, -1.62], Hedges’s g = -2.48; opposite: t = -3.23, p-value = 0.008, 95% CI [-1.67, -0.56], Hedges’s g = -1.09). Among all participants, as expected, the condition with the lowest performance was when target and reference speeds were equal (coherent vs spatial: t = 4.33, p-value < 0.001, 95% CI [-2.27, -0.94], Hedges’s g = -1.57; coherent vs temporal: t = 12.66, p-value < 0.001, 95% CI [-3.45, -1.62], Hedges’s g = -2.48; coherent vs opposite: t = 9.97, p-value < 0.001, 95% CI [1.26, 2.82], Hedges’s g = 2.00). The last comparison between conditions showed that participants were significantly less precise in the spatial condition than in the opposite condition (t = 7.57, p-value <0.001, 95% CI [1.00, 2.38], Hedges’s g = 1.66), suggesting that participants’ performance was lower when temporal information were not available for speed evaluation (as in the spatial condition in which target and reference sound had the same temporal duration), compared to when spatial but also temporal cues were available, and the temporal information could help participants (as in the opposite condition). It is worth specifying that the temporal properties were successfully informative only when applying the temporal assumption that short duration corresponds to fast speed was true (i.e. in the temporal and opposite condition), and not when this temporal assumption was not satisfied (i.e., in the coherent and spatial condition). Finally, a significant interaction between Group and Condition (Iter: 5000, F = 11.79, p-value < 0.001, 95% CI [0.12, 0.45], partial *η*^2^ = 0.31) suggested that participants’ performance was influenced by their visual experience, but also by the kind of cues that, according to the experimental condition, were available for speed discrimination (Fig 7). Post-hoc comparisons were conducted with permutation paired t-tests, and Bonferroni-corrected for multiple comparisons. EB individuals were significantly less precise than SC in the spatial and coherent conditions (spatial: t = 3.88, p-value = 0.006, 95% CI [0.63, 2.50], Hedges’s g = 1.50; coherent: t = 6.73, p-value = 0.001, 95% CI [0.72, 2.70], Hedges’s g = 1.64), while in the temporal and opposite conditions the two groups reported a similar performance (temporal: t = 0.34, p-value = 1, 95% CI [-0.52, 0.68], Hedges’s g = 0.08; opposite: t = 0.95, p-value = 1, 95% CI [-0.36, 0.85], Hedges’s g = 0.24). The conditions that revealed a greater difference between the EB and SC participants (spatial and coherent conditions), also inverted the psychometric curves (see Fig 4 for an example) and indicated that participants were impeded when performing the speed discrimination task because in these conditions following the temporal assumption was misleading. EB participants were so attracted by the temporal aspects of motion that they rely on them even when they were incorrect, more than SC. This result is peculiar because it suggests that the lack of vision may play a role in the erroneous use of temporal information for motion processing.

## Discussion

The present study investigated how visual experience shapes spatial and temporal representations underlying acoustic speed perception. An array of speakers reproduced sounds traveling at different speeds, and early blind and sighted participants had to determine how fast they were moving by relying on duration, travelled distance, or both. We investigated whether visual deprivation could lead to an improvement in the acoustic speed perception (i.e., possibly due to neural plasticity), or an impairment due to the lack of visual calibration on acoustic speed perception. As hypothesized, we observed the sound speed discrimination of the early blind participants being influenced by a peculiar processing of spatio-temporal motion cues (duration and distance) that led to a worst performance. Indeed, our results showed that the early blind individuals, more than the sighted controls, were unable to discriminate the sound speed when no temporal information was provided (i.e., in the spatial condition) and when they erroneously applied a temporal assumption associating fast speeds with short sound durations (i.e., in the coherent condition). Overall these results suggest that the impact of the visual deprivation on auditory motion perception is related to a distinctive use of spatio-temporal information by early blind individuals.

The present study showed a temporal attraction of both sighted and blind participants, which led only blind individuals to be specifically impaired in their auditory speed perception. On what concerns all participants’ temporal attraction in the speed discrimination, this was expressed by considering two main aspects. First, the temporal components of moving sounds dominated the speed discrimination performance in both sighted and early blind individuals (i.e., temporal dominance). This result confirmed past findings on sighted adults that challenged the existence of acoustic motion-sensitive detectors similar to those in the visual system [1, 4]. However, this was the first study showing auditory motion’s temporal dominance in the visually impaired population. Previous studies had already revealed the good performance of blind individuals in temporal tasks, occasionally expressed as superior temporal abilities than sighted controls [18–20]. In addition to this evidence, we showed that the lack of vision does not prevent the temporal preference in acoustic motion, suggesting that blindness does not allow the recruitment of visual motion-sensitive detectors by audition, as one could hypothesize in a cross-modal cortical reorganization point of view. However, further investigations in this direction are needed. The second aspect of the temporal attraction was all participants considering speeds as faster every time the target sound was shorter in time independently of space, suggesting a temporal assumption. By following this temporal assumption, participants showed a good speed discrimination performance in the experimental conditions wherein shorter sound durations did actually imply faster speeds. This was the case of the temporal condition (where only temporal information was available) and the opposite condition (where the temporal assumption could be applied to time, independently of space).

On the contrary, in the experimental conditions wherein shorter sound durations did not imply faster speeds, the temporal assumption application led participants to misperceive the actual speeds of the sounds. For example, in the coherent condition, although speed was invariable between the target and reference sounds, considering the temporal assumption gave listeners the impression that a change in sound duration meant a change in velocity between the two sounds. Visual experience does not seem to be a prerequisite in gaining the temporal assumption since both early blind and sighted participants reported this aspect. Even though further investigation is needed, the temporal assumption we revealed might be attributed to previous knowledge that reflects the probability distribution of the environment’s speed property [44–46]. Environmental statistics help humans to predict the events of the world. We therefore speculate that relating fast speeds to short durations may be a preventive way to react rapidly to stimuli that are approaching quickly. Although we suggest further investigation in this direction, the temporal attraction we describe herein confirmed the close relationship between the auditory system and the temporal domain and showed that this connection exceeds the properties-matching between the two aspects, also touching higher levels of one’s world understanding.

Despite the similarities in temporal attraction between the early blind and the sighted groups, we showed that the visual experience plays a fundamental role in perceiving speed through audition. Indeed, the second main result of the present study is early blind participants revealing a worse performance than sighted controls in discriminating the moving sounds’ speed. Specifically, the significant difference between the groups emerged in two experimental conditions. In the spatial condition, to discriminate the sounds’ speed, participants could rely exclusively on distance cues. One may therefore attribute the impaired performance of the early blind to the spatial deficit of this population that other studies have outlined [21, 22]. Moreover, previous research suggested that when blind individuals face spatial tasks that they cannot solve, they tend to rely on the event’s temporal aspects [30, 31]. The brain may assume a prior of stimulus’ constant velocity that could represent a channel of communication between space and time domains (for more details on the Imputed Velocity Theory see [47]). It has been suggested that blind individuals rely more on the constant velocity prior in order to use the unimpaired temporal coordinates to decode the spatial metrics [48]. We propose that, in our study, the process of using time to infer space showed up in blind participants who applied, more than the sighted controls, the temporal assumption of short duration always associated with fast speed, to the spatial domain. Specifically, participants erroneously interpreted smaller traveled distances as quicker movements, similarly to how they interpreted duration in the temporal condition, even if in the spatial condition the assumption short corresponding to fast was invalid. In the coherent condition instead, the target sound speed was equal to the reference sound speed, with distance cues proportional to duration cues (see Fig 2 for more details). Given the correspondence of the spatial and temporal information, it might be possible that visually impaired individuals selectively focused their attention on the domain they encode better (i.e., time) and neglected the presence of spatial information. In the coherent condition the misleading focus on the stimulus’s temporal aspects may therefore explain the lower precision of early blind participants in the speed domain.

A limitation of the current work was the small sample size that constrained the results’ generalization, even if sharing a similar number with other studies involving early blind individuals [21–24, 28]. Future studies using larger sample sizes should be implemented, also involving late blind and low-vision participants for a more in-depth investigation.

To conclude, our results showed that the absence of visual experience early in life might influence the ability to encode the speed of sounds in motion by increasing the preference of blind individuals towards the stimuli’ temporal characteristics. Both sighted and early blind individuals reported this temporal attraction. We speculate that this process might be mediated by a lack of auditory motion-sensitive detectors in both populations. However, when the temporal preference was misleading, the speed perception of participants with visual disabilities was more affected. This observation suggests that an early multisensory perception of the world is crucial to prevent the misleading use of compensatory behaviors, which can turn into maladaptive practices if generated by unsuccessful environmental sampling and erroneous top-down predictions (for a review on the topic, see [49]). Assessing the presence of maladaptive compensatory behaviors and evaluating the functioning of blind individuals is extremely important to consider, especially with reference to design rehabilitation programs for this population. For example, new rehabilitative strategies could mitigate temporal attraction during speed encoding when non-functional.
